# Improved Appropriateness of Advanced Diagnostic Imaging After Implementation of Clinical Decision Support Mechanism

**DOI:** 10.1007/s10278-021-00433-6

**Published:** 2021-02-25

**Authors:** Leonid L. Chepelev, Xuan Wang, Benjamin Gold, Clara-Lea Bonzel, Frank Rybicki Jr, Jennifer W Uyeda, Adnan Sheikh, Dan Anderson, Jared Lindaman, Greg Mogel, Dimitrios Mitsouras, Mary C. Mahoney, Tianxi Cai, Frank J. Rybicki

**Affiliations:** 1 Stanford University, Stanford, CA USA; 2 Harvard School of Public Health, Boston, MA USA; 3 Change Healthcare, Madison, WI USA; 4 Harvard Medical School, Boston, MA USA; 5 University of Ottawa, Ottawa, Ontario Canada; 6 University of California San Francisco, San Francisco, CA USA; 7 University of Cincinnati, Cincinnati, Ohio USA

**Keywords:** Appropriateness Criteria, Big Data, Centers for Medicare and Medicaid Services, Clinical Decision Support, Compliance, Electronic Medical Record, Imaging Appropriateness, Imaging Metrics, Medical Imaging, Protecting Access to Medical Care, Qualified Provider Led Entity, Radiology

## Abstract

The Protecting Access to Medicare Act (PAMA) mandates clinical decision support mechanism (CDSM) consultation for all advanced imaging. There are a growing number of studies examining the association of CDSM use with imaging appropriateness, but a paucity of multicenter data. This observational study evaluates the association between changes in advanced imaging appropriateness scores with increasing provider exposure to CDSM. Each provider’s first 200 consecutive anonymized requisitions for advanced imaging (CT, MRI, ultrasound, nuclear medicine) using a single CDSM (CareSelect, Change Healthcare) between January 1, 2017 and December 31, 2019 were collected from 288 US institutions. Changes in imaging requisition proportions among four appropriateness categories (“usually appropriate” [green], “may be appropriate” [yellow], “usually not appropriate” [red], and unmapped [gray]) were evaluated in relation to the chronological order of the requisition for each provider and total provider exposure to CDSM using logistic regression fits and Wald tests. The number of providers and requisitions included was 244,158 and 7,345,437, respectively. For 10,123 providers with ≥ 200 requisitions (2,024,600 total requisitions), the fraction of green, yellow, and red requisitions among the last 10 requisitions changed by +3.0% (95% confidence interval +2.6% to +3.4%), −0.8% (95% CI −0.5% to −1.1%), and −3.0% (95% CI 3.3% to −2.7%) in comparison with the first 10, respectively. Providers with > 190 requisitions had 8.5% (95% CI 6.3% to 10.7%) more green requisitions, 2.3% (0.7% to 3.9%) fewer yellow requisitions, and 0.5% (95% CI −1.0% to 2.0%) fewer red (not statistically significant) requisitions relative to providers with ≤ 10 requisitions. Increasing provider exposure to CDSM is associated with improved appropriateness scores for advanced imaging requisitions.

## Introduction

The Protecting Access to Medicare Act (PAMA) of 2014, Section 218(b) [1] established a new program to increase appropriateness for advanced diagnostic imaging that requires [2] ordering providers to consult a qualified Clinical Decision Support Mechanism (CDSM) for Medicare beneficiaries. The Centers for Medicare and Medicaid Services (CMS) may collect data on these transactions and may penalize noncompliant requisitions. Implementing Clinical Decision Support (CDS) has many challenges, including accurate quantification of the overall effect of CDS implementation and identification of key markers of success such as the levels of inappropriate high-cost imaging [3]. This observational study provides analyses of a large dataset collected after CMS convened the Demonstration Project (2011–2013) [4] and before the implementation of the CDSM mandate, and specifically tests the hypothesis that there is an association between medical imaging CDS and appropriateness scores at the requisition provider level.

Among other requirements [2], for each consultation the CDSM must determine and generate documentation on whether the service ordered would or would not adhere to Appropriate Use Criteria (AUC) or whether the AUC consulted was not applicable to the service ordered. Some qualified Provider-Led Entities (qPLE) categorize requisitions into “usually appropriate” (green), “may be appropriate” (yellow) and “rarely appropriate” (red) for specific clinical scenarios. For this project, gray requisitions were defined as those not assigned to an AUC. CMS lists over 20 qPLEs [5] including, for example, organizations such as the American College of Radiology (ACR) and the American College of Cardiology, as well as universities. Each of these organizations must apply for and maintain an active qPLE status with CMS. In terms of the total number of AUCs, the largest qPLE is the ACR. The AUC logic is incorporated into CDSM that are typically integrated into electronic medical record (EMR) systems. The software may intervene at the level of order entry to provide immediate feedback and can provide long-term trends on order appropriateness for specific clinical scenarios submitted by a specific requisition provider.

Several studies examining the impact of CDS implementation on imaging appropriateness have been performed. The largest study on the subject to date, known as the Demonstration Project [4], has examined a total of 117,348 requisitions, of which up to 66.5% were “gray” requisitions as they had no corresponding records of structured clinical scenarios mapped to corresponding appropriateness scores. The remaining structured and mapped requisitions in this study demonstrated a 7.3% increase in appropriate studies and a 4.7% decrease in inappropriate studies. Due to limited understanding of gray requisitions, the true scale of appropriateness changes was not well characterized. This limitation prompted authors to consider alternative ways to examine the impact of CDS, including by conducting randomized trials at an institutional level [6] or by conducting retrospective analyses on smaller sets of clinical scenarios to quantify associations between CDS among specific uses, for example for a relatively narrow specialty [7].

The most recent and most comprehensive prospective randomized controlled trial [6] divided 3524 ordering providers at a single medical center into approximately equal groups of providers with access to a CDSM integrated into an EMR at the time of order entry and those using traditional order entry techniques without CDS access. Both groups used the same software environment, but the participants of the CDS group were provided with immediate automated feedback with notifications regarding inappropriate orders. Following an initial 8-month observation period where no notifications were shown to either group, but data was collected, notifications were turned on for the CDS group for a period of 12 months. Over a period of 12 months, the CDS group demonstrated a 6% decrease in the sum of red and yellow requisitions but no significant change in high- or low-cost scans, supporting a modest positive impact of CDS implementation.

Similar randomized controlled trials with matched groups would be exceedingly difficult to coordinate on a national level, as CDSM are almost always localized to particular institutions. Moreover, since the implementation of the PAMA mandate, many organizations that might otherwise be interested in randomized trials have already established a CDSM to be compliant. At the same time, a better understanding of an association of CDS implementation with appropriateness is highly desirable as an initial means to infer costs, and ideally, such analyses would span a heterogeneous set of institutions with varied cultures, attitudes towards CDS, policies, and software environments (among other factors). Observational retrospective studies thus provide the most rigorous multicenter analyses currently available. Such multicenter and national level assessments most frequently use “time since intervention” to benchmark changes in imaging appropriateness. For example, while the Demonstration Project [4] considered multiple heterogeneous organizations in 8 states, changes in image requisition patterns were considered on the aggregate population. While convenient, such an approach assumes complete homogeneity of the aggregate provider population and stability of external factors impacting that population over the period of assessment. While this assumption may be relatively easy to maintain with a limited and constant number of centers, assessments evaluating national-level data are more complex. For example, one must consider numerous factors such as the total number of centers available within a database at a given time, changes in software content between progressive versions, variability in how the CDSM is implemented in terms of scope and workflow, local hospital policy changes, educational environments, complexity of patient care, patient demographics, and the levels of staff turnover, among others. Each of these factors can potentially reduce reproducibility when considering the impact of CDS on requisition appropriateness on provider level.

An important premise for the current project is that learning may occur with every provider-CDSM interaction, and that the effect could vary in proportion to the total number of provider-CDSM interactions due to reinforcement, in line with basic operant conditioning learning [8]. The methodology we choose to evaluate the association between appropriateness scores and CDSM exposure relates directly to exposure or the number of times that a provider orders a high cost imaging study. As an example, a provider exposed to a year of CDSM who has submitted three requisitions in that time has likely learned much less from the CDSM than another provider who submits 30 requisitions in a single month.

Our approach uses provider-level data and the proportion of green, yellow, and red rates to benchmark CDSM impact because this metric can be applied individually, or it can be aggregated up to a national level. To assess the relationship between CDSM exposure and imaging appropriateness quantitatively, this study leverages large-scale data on provider CDSM exposure and the proportion of green, yellow, red, and gray appropriateness scores across the USA.

## Materials and Methods

In this observational study, all computed tomography, magnetic resonance, ultrasound, and nuclear medicine consecutive requisition data collected from the CareSelect^™^ (Change Healthcare, Madison, WI) CDSM between January 1, 2017 and December 31, 2019 were de-identified and sent for independent evaluation at the University of Cincinnati, where a waiver for written informed consent was obtained. Statistical analyses were independently performed at the Department of Biostatistics, Harvard School of Public Health (Boston, MA). No data was evaluated by any employee at Change Healthcare, and none of the other authors have ever been employed or received any funding whatsoever from Change Healthcare or any other CDSM, any qPLE, or any company related to Electronic Medical Records. Requisitions from providers mapped to an AUC other than the ACR were excluded because they represented a minority (7.1%) of the sampled requisitions and were implemented heterogeneously. Providers not mapped to a unique anonymized identifier in the CDSM were excluded. To minimize impact from providers with CDSM exposure before 2017, individuals with first observed requisitions in January 2017 were excluded. The data spanned three versions of the CDSM implemented within the electronic medical record (EMR) at 288 institutions. All authors who analyzed the data were blinded to the identity of the sites and individual providers.

At the provider level, we examined changes in the proportion of green, yellow, red, and gray requisitions (ordinates) to identify a potential relationship between CDSM and ordering appropriateness scores. The hypothesis that there is an association between medical imaging CDS and appropriateness scores at the requisition provider level was tested using several analyses.

In a preliminary descriptive assessment, for each provider, all requisitions were arranged in chronological order from requisition 1 through a cutoff of 200 chosen to plot early evolution of appropriateness following CDS exposure. The abscissa contained 20 chronological groups of 10 requisitions each; requisitions 1–10 were placed in group 1, 11–20 in group 2, and 191–200 in the final group. Only summary statistics were derived.

The first analysis included providers with 200 requisitions or more using a multinomial logistic regression fit on requisition-level data to estimate how the probability of a requisition being green, yellow, red, or gray varied with chronological requisition order. We used cubic spline basis with three knots for the chronological order to capture potential non-linear trends based on data review. Statistical significance of the changes in the distribution of appropriateness categories in relation to the first group was assessed based on Wald tests from the logistic fits.

In the second analysis, each provider was assigned an “experience rank” defined as their total number of submitted requisitions. The abscissa contained 20 groups, with 10 sequential experience rankings each. Providers with a rank of 201 and above were discarded with the same rationale as in the first analysis. A quasi-binomial logistic regression model was fit to assess the association between provider experience ranking and their total percentages of green, yellow, red, and gray requisitions. We used cubic spline basis with three knots for the experience ranking to incorporate non-linear effects. The statistical significance was assessed based on Wald tests on the percentages of green, yellow, red, and gray requisitions as a function of the 20 experience ranking bins.

## Results

A total of 268,095 providers created 16,497,300 requisition modification records. Of these, 1,893,378 records with AUC not mapped to ACR guidelines were excluded. An additional 59,217 records were excluded because there was no unique provider ID. The remaining 14,544,705 records were mapped to 12,154,127 unique requisitions submitted by 258,136 providers with an overall appropriateness score breakdown of 56.9% (6,910,194) green, 15.9% (1,933,130) yellow, 12.8% (1,550,160) red, and 14.5% (1,760,643) gray. Of these, 7,345,437 requisitions (60.4%) had a chronological index less than 201 and were submitted by 244,158 providers starting on February 1, 2017; these were included in the descriptive assessment. A total of 10,123 providers submitted at least 200 requisitions each, for a total of 2,024,600 requisitions included in the analysis of the correlation between the number of submitted requisitions and requisition appropriateness. There were 234,035 (87.3%) providers who submitted fewer than 201 requisitions each and were included in the analysis of the correlation between the total provider CDSM exposure and total per-provider requisition appropriateness, with 5,320,103 requisitions.

**Fig. 1:**
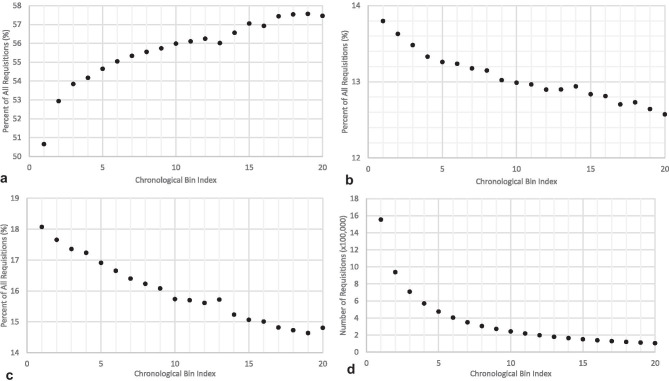
Observational data for the distribution of the green **a**, red **b**, and yellow **c** requisitions in chronological bins indexed by 10 requisitions per bin, and the absolute number of requisitions in each bin **d**

In the observational data (Fig. 1), the green rate varied from 50.7% in the lowest chronological bin index to 57.5% in bin 20, the yellow rate from 18.1 to 14.8%, the red rate from 13.8 to 12.6%, and the total number of requisitions from 1,554,847 to 104,997.

**Fig. 2:**
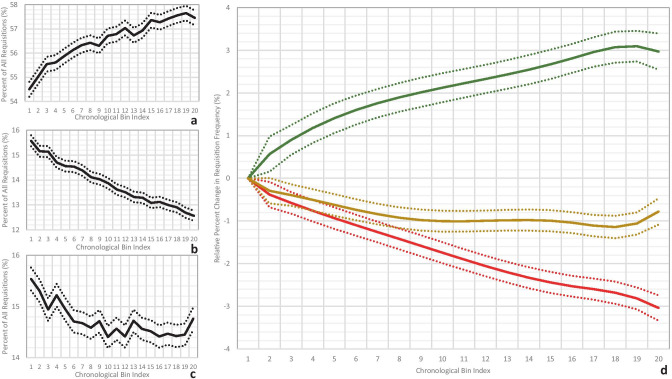
Absolute percent changes in the percent distribution of green **a**, red **b**, and yellow **c** requisitions over the chronological bin indices (solid lines) with associated standard deviations (dotted lines) and for all providers with at least 200 requisitions. **d** Generalized linear model fits for the green, red, and yellow requisition percentage changes (color-mapped, respectively), in comparison with the first category as baseline, with 95% confidence intervals (dotted lines)

In the first analysis (Fig. 2), the fraction of green requisitions increased from 54.5 to 57.5% with increasing CDS exposure, the yellow requisitions decreased from 15.5 to 14.8%, and the red requisitions decreased 15.6% to 12.6%. Thus, the fraction of green, yellow, and red requisitions among the last 10 requisitions changed by +3.0% (95% confidence interval +2.6% to +3.4%), −0.8% (95% CI −0.5% to −1.1%), and −3.0% (95% CI 3.3% to −2.7%) in comparison with the first 10, respectively.

**Fig. 3:**
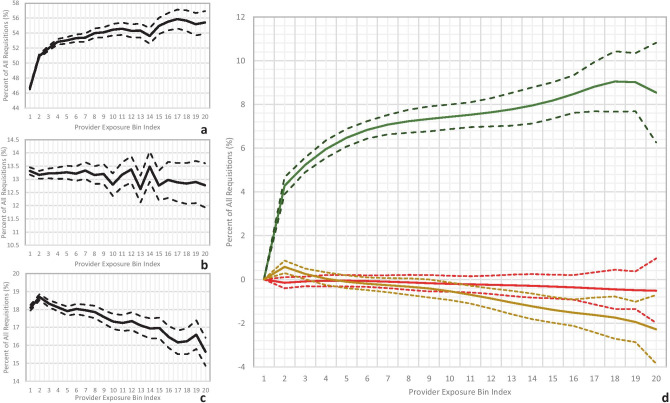
Absolute percent changes in the percent distribution of green **a**, red **b**, and yellow **c** requisitions over the provider experience category (solid lines) with associated standard deviations (dotted lines). **d** Generalized linear model fits for the green, red, and yellow requisition percentage changes (color-mapped, respectively), in comparison with the first category as baseline, with 95% confidence intervals (dotted lines)

The second analysis (Fig. 3) demonstrated similar trends across provider experience ranks. Providers with > 190 requisitions had 8.5% (95% CI 6.3% to 10.7%) more green requisitions, 2.3% (0.7% to 3.9%) fewer yellow requisitions, and 0.5% (95% CI −1.0% to 2.0%) fewer red (not statistically significant) requisitions relative to providers with ≤ 10 requisitions. All changes were significant at *p* < 0.05 level except the decrease in fraction of red requisitions across total provider experience.

## Discussion

Medical imaging CDSM use was associated with an overall improvement in appropriateness scores as measured by the chronology of requisitions and the experience of the provider. Under the assumption that appropriateness scores rendered by the CDSM at the point of care correlate with an improvement in imaging appropriateness, the improved green and red rates support CDSM consultation for high-cost medical imaging.

While there is general agreement that overuse of advanced diagnostic imaging contributes to the high cost of healthcare in the USA, uncertainty remains about the effectiveness of CDS in improving quality or reducing costs. This study benchmarks appropriateness scores for contemporary medical imaging CDS implementation in the USA for one AUC across a large scope of providers, and when compared with the Demonstration Project and other early CDS studies [9] shows an overall lower gray rate. Another strength is that the potential benefit of using a CDSM is studied in two ways, by assessing the total requisition provider exposure and studying the evolution of requisitioning practices chronologically.

Limitations of our retrospective, observational study include a lack of data prior to CDS implementation; ideally, the observational study would include data from before implementation of a CDSM. While we fully recognize this limitation, to our knowledge, this data is not available at a larger scale level. We also acknowledge that our study cannot definitively identify CDSM use as the causative factor in appropriateness changes. Hospitals often introduce other initiatives to reduce high-cost scanning procedures [6]. We admit that we are unable to follow and control for these factors. An additional limitation is the assumption that providers with CDSM prior to January 2017 would have a documented transaction during January 2017. Finally, we acknowledge that we have only considered data from one qPLE and we have only studied a single CDSM, when in fact there are many of each in clinical practice.

Regarding the clinical impact of this initial evaluation, one should challenge the assumption that appropriateness scores rendered by the CDSM at the point of care correlate with an improvement in imaging appropriateness. A more pessimistic and critical stance suggests “some learning has been demonstrated, but that learning could be entirely gaming the CDSM with no proven impact on imaging decisions whatsoever”. Formally, we acknowledge that the benefit seen in appropriateness scores from this project could be entirely from gaming. Conversely, a more optimistic and naïve stance might suggest “all scores reflect imaging decisions and imaging appropriateness is improved across the board in the US after CDSM is implemented.” We believe that the truth likely lies between these two viewpoints.

We recognize two potential gaming strategies. In the first, a provider may attempt to bypass formally specifying poorly scoring requisitions by using unmappable text such as “.” or “!”. This is expected to increase the gray rate with comparable decreases in red rates. The second strategy is selection of known “green” indications that inaccurately describe a clinical scenario. One example would be collapsing all patients with abdominal pain to “suspected renal calculi”, since it maps to a “known green” appropriateness score for CT. The pool of tactics for the second strategy is expected to be limited, and overall would result in a fall in requisition diversity.

We performed a subanalysis of CT requisitions with uniquely identified but not ontologically mapped clinical scenarios for all 2,748 providers with more than 200 CT requisitions but fewer than 400 total requisitions. We compared the first 10 (total 27,480) requisitions to the last 10 (total 27,480) requisitions in a 200-requisition per-provider sample using methods defined in the first analysis. The changes in green: 58.2% (57.2–59.1%) to 62.1% (61.1–63.1%), yellow: 15.6% (15.0–16.2%) to 13.8% (13.2–14.4%), red: 15.3% (14.6–15.9%) to 12.3% (11.7–12.9%), and gray: 10.9% (10.3–11.6%) to 11.8% (11.0–12.6%) rates in this sample (95% confidence intervals provided in brackets) were statistically significant with a *p* < 0.02 on a paired two-tailed Student’s *t* test. This recapitulates trends in the overall data of 10,123 providers, where the overall +0.7% change in the gray rate also does not account for the sum of other improvements in appropriateness scores over the observed CDS exposure, arguing against the first gaming strategy as a major driver of overall appropriateness score changes.

In the same subanalysis, the number of requisition categories increased by 92 between the two bins (1,765 in bin 1 to 1,857 in bin 20), corresponding to a higher Shannon diversity index of 5.83 among the last 10 requisitions relative to 5.72 in the first 10 (Hutcheson *t* test, *p* ≪ 0.01). Shannon equitability indices, which describe the degree of separation of the sample from a perfectly equitably distributed set of categories (at index of 1), were 0.765 and 0.774 for the first and last bins, respectively. This increase in requisition diversity argues against a significant contribution of the second gaming strategy to overall score dynamics.

While this preliminary subpopulation assessment offers limited insight on gaming, more comprehensive study is required. One next step will include recruitment of independent experts to adjudicate appropriateness using complete patient electronic health records and imaging reports, a topic outside of the scope of this investigation due to limitations on available patient information.

While larger than previous imaging AUC studies, with increasing PAMA compliance, our current database is expected to be modest in comparison with future research. This early report will be followed by studies to provide insight on the evolving priorities in medical imaging [10]. More comprehensive, future data could prove beneficial for qPLEs and, where applicable, guideline committees who generate AUC. The project will evolve in at least two directions. First, additional attention will focus on metrics that can be used to test the proportion of requisitions considered green, yellow, red, and gray. Second, we will continue to accrue and evaluate additional data to dissect the features of providers and requisitions for hypotheses testing, for example the distribution of CDS events based on provider specialty and clinical setting (e.g., emergency department versus inpatient versus outpatient). CMS has also identified Priority Clinical Areas as important for future study, and thus, appropriateness for these specific scenarios can be queried. Future work could also assess the impact of CDSM exposure on the use of high cost imaging studies, and if requisitions considered appropriate demonstrated a higher positivity rate for identification of disease states that altered clinical care. Testing the later hypothesis would require linking individual requisitions to a set of clinical outcomes.

The gray rate decreased from almost two-thirds in the Demonstration project to approximately 15% in our study. Specific attention to these 1,760,643 gray requisitions—including analytics of free text entries—may provide important insights for future investigations. The present study provides the largest data set to our knowledge to help understand an association between the use of a CDSM and advanced imaging order appropriateness scores. By tracking and aggregating changes in appropriateness scores among providers as a function of CDSM exposure, this study is able to address some inherent limitations from large, heterogeneous data. Analyses that use larger sample sizes will likely be less exposed to random sampling error due to practice variability. Alternative time-based metrics do not accurately capture the true extent of CDSM exposure as a result of practice and provider variability in ordering high-cost imaging studies. It has been suggested that inappropriate high-cost imaging is concerning from the perspective of health risks and healthcare costs [6]. Strategies should impact individual provider behavior to optimize care quality, safety, and healthcare economics. The proposed metrics used to test the hypothesis that there is an association between medical imaging CDS and appropriateness at the requisition provider level can also be scaled to benchmark departmental, specialty-wide, organizational, regional, and national-level performance.

## Conclusion

The positive association of increased CDSM exposure with improved requisition appropriateness scores supports CDSM consultation for high cost imaging. Future work should correlate appropriateness scores with pragmatic clinical appropriateness and outcomes, and further analyses at individual provider and individual requisition level are planned. Future work will also identify the specialties, provider characteristics, and clinical scenarios where targeted interventions may be leveraged to improve imaging appropriateness and potentially the overall quality and safety of medical imaging.
